# Supplementary material for: Incidence patterns of orofacial clefts in purebred dogs
Source: PLoS One. 2019 Nov 4;14(11):e0224574. doi: 10.1371/journal.pone.0224574 (PMC6827884; doi:10.1371/journal.pone.0224574)
Supplement: S1 File — Copy of the online survey used to collect the data reported in this study. (PDF) [file pone.0224574.s001.pdf]

Introduction and Consent

We are asking you to participate in a research study on the incidence of orofacial clefts (cleft lip, cleft palate, or both) at birth in several dog breeds. You will be asked to fill out a survey about litters you have whelped in the past year.

Purpose of the study: To determine whether certain purebred dogs have a higher risk of orofacial clefts and to estimate the financial impact on breeding programs.

Time commitment: The survey should generally take 10 to 20 minutes to complete (it may take longer for extremely large breeding programs). No personally identifiable information is collected as part of the survey.

What you get out of it: Getting involved in research is a powerful way to be a part of veterinary medical breakthroughs. While we cannot offer financial compensation for participating in this survey, we hope that the research may eventually help to improve breeding programs. By participating in this study, you can help improve researchers' understanding of the frequency with which orofacial clefts occur as well as possible causes, predictors, and risk factors. The information gained may potentially help veterinarians and researchers to diagnose, treat, and prevent orofacial clefts and thus improve breeding operation economics.

This study is being performed by professor Santiago Peralta, DVM, DAVDC, and other colleagues in the Department of Clinical Sciences at Cornell University's College of Veterinary Medicine. Should you have questions or concerns about the study, Dr. Peralta can be contacted via email at sp888@cornell.edu.

Participation in this survey is voluntary. All participants must be at least 18 years old. You may refuse to participate, discontinue at any time, or skip any questions, with no penalty, although incomplete surveys may not be included in the study

analysis. If you have any questions or concerns regarding your rights as a subject in this study, you may contact the Institutional Review Board (IRB) for Human Participants at 607-255-6182 or access their website at http://www.irb.cornell.edu. You may also report your concerns or complaints anonymously through Ethicspoint online at www.hotline.cornell.edu or by calling toll free at 1-866-293-3077. Ethicspoint is an independent organization that serves as a liaison between the University and the person bringing the complaint so that anonymity can be ensured.

I have read the introduction to the survey and consent to take part in the study.

- Yes
- No

Survey Questions

Section 1: Breeding program

Question 1: What breed of dog do you produce in your breeding operation? If you breed multiple breeds, please fill out the survey once per breed.

- Boston Terrier
- Boxer
- English Bulldog
- French Bulldog
- Beagle
- Golden Retriever
- Jack Russell Terrier
- Labrador Retriever
- Collie
- Dachshund
- Doberman Pinscher
- Italian Greyhound

Question 2: Which of the following geographic regions is the breeding operation located in?

- Northeast (Connecticut, Maine, Massachusetts, New Hampshire, New Jersey, New York, Pennsylvania, Rhode Island, and Vermont)
- Midwest (Iowa, Illinois, Indiana, Kansas, Michigan, Minnesota, Missouri, North Dakota, Nebraska, Ohio, South Dakota, and Wisconsin)
- South (Alabama, Arkansas, District of Columbia, Delaware, Florida, Georgia, Kentucky, Louisiana, Maryland, Mississippi, North Carolina, Oklahoma, South Carolina, Tennessee, Texas, Virginia, and West Virginia)
- West (Alaska, Arizona, California, Colorado, Hawaii, Idaho, Montana, New Mexico, Nevada, Oregon, Utah, Washington, and Wyoming)
- Other

Question 3: In your breeding operation, how many litters did you whelp in the past 12 months?

Question 4: How many live births (total number of live puppies) did you whelp in the past 12 months?

Section 2: Types of orofacial clefts

This study is concerned with the live-birth incidence of orofacial clefts. Please provide information pertaining to puppies born alive, rather than stillborn puppies.

Orofacial clefts are diagnosed in affected puppies by visual inspection of the face and

oral cavity. Orofacial clefts can be categorized as **cleft lip** only, **cleft palate** only, or **both** cleft lip and cleft palate (see images and definitions below).

Cleft Lip Only--Lip defects on one or both sides without hard or soft palate defects

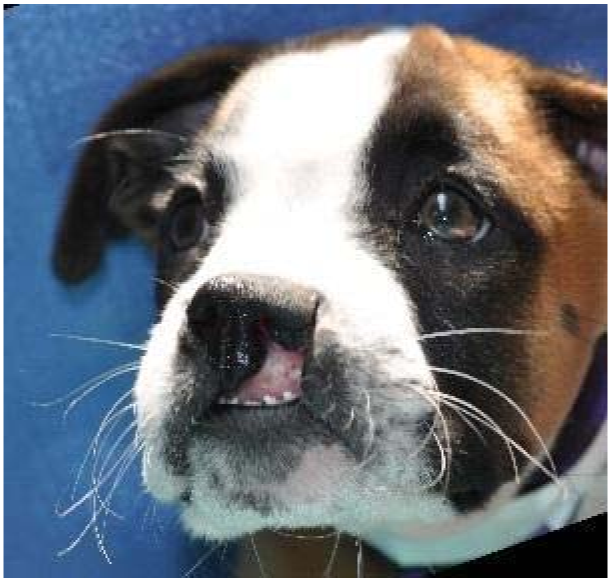

Cleft Palate Only--Defects affecting only the hard and soft palate but not the lips

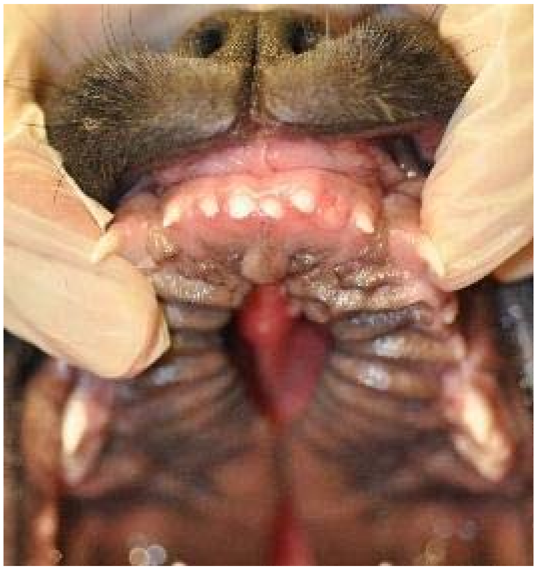

Cleft Lip and Palate--Defects affecting the lip on one or both sides, as well as the hard and soft palates

Question 5: In the past 12 months, did you have any litters that included one or more live puppies with an orofacial cleft (cleft lip, cleft palate, or both)?

- Yes
- No

Section 3: Numbers of orofacial clefts

This study is concerned with the live-birth incidence of orofacial clefts. Please provide information pertaining to puppies born alive, rather than stillborn puppies.

Question 6: Of the litters you whelped in the past 12 months, how many litters included one or more live puppies with an orofacial cleft (cleft lip, cleft palate, or both)?

- 1
- 2
- 3
- 4
- 5
- 6
- 7
- 8 or more

Section 4: Cleft lip only

This study is concerned with the live-birth incidence of orofacial clefts. Please provide information pertaining to puppies born alive, rather than stillborn puppies.

Question 7: How many live puppies had **cleft lip only** in the past 12 months?

- 0
- 1
- 2
- 3
- 4
- 5
- 6
- 7
- 8 or more

How many of the affected puppies were male?

- 0
- 1
- 2
- 3
- 4
- 5
- 6
- 7
- 8 or more

How many of the affected puppies were female?

- 0
- 1
- 2
- 3
- 4
- 5
- 6
- 7
- 8 or more

Please indicate if these were diagnosed by a veterinarian or yourself.

- Veterinarian
- Myself
- Other

Please indicate if any other congenital abnormalities were also present, such as skeletal malformations.

- Yes
- No

Cleft Lip Only--Lip defects on one or both sides without hard or soft palate defects

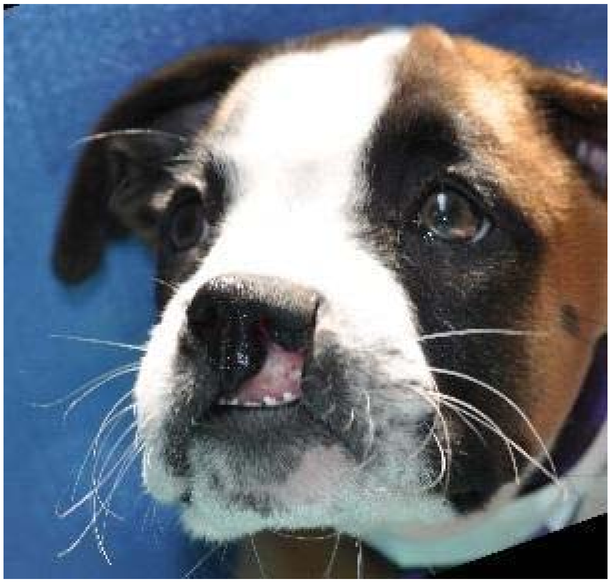

Section 5: Cleft palate only

This study is concerned with the live-birth incidence of orofacial clefts. Please provide information pertaining to puppies born alive, rather than stillborn puppies.

Question 8: How many live puppies had **cleft palate only** in the past 12 months?

- 0
- 1
- 2
- 3
- 4
- 5
- 6
- 7
- 8 or more

How many of the affected puppies were male?

- 0
- 1
- 2
- 3
- 4
- 5
- 6
- 7
- 8 or more

How many of the affected puppies were female?

- 0
- 1
- 2
- 3
- 4
- 5
- 6
- 7
- 8 or more

Please indicate if these were diagnosed by a veterinarian or yourself.

- Veterinarian
- Myself
- Other

Please indicate if any other congenital abnormalities were also present, such as skeletal malformations.

- Yes
- No

Cleft Palate Only--Defects affecting only the hard and soft palate but not the lips

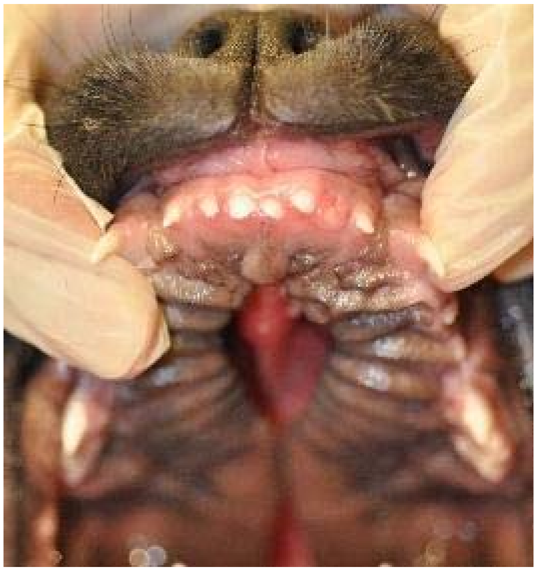

Section 6: Both cleft lip and cleft palate

This study is concerned with the live-birth incidence of orofacial clefts. Please provide information pertaining to puppies born alive, rather than stillborn puppies.

Question 9: How many live puppies had **both cleft lip and cleft palate** in the past 12 months?

- 0
- 1
- 2
- 3
- 4
- 5
- 6
- 7
- 8 or more

How many of the affected puppies were male?

- 0
- 1
- 2
- 3
- 4
- 5
- 6
- 7
- 8 or more

How many of the affected puppies were female?

- 0
- 1
- 2
- 3
- 4
- 5
- 6
- 7
- 8 or more

Please indicate if these were diagnosed by a veterinarian or yourself.

- ○ Veterinarian
- ○ Myself
- ○ Other [ ]

Please indicate if any other congenital abnormalities were also present, such as skeletal malformations.

- ○ Yes [ ]
- ○ No

Cleft Lip and Palate--Defects affecting the lip on one or both sides, as well as the hard and soft palates

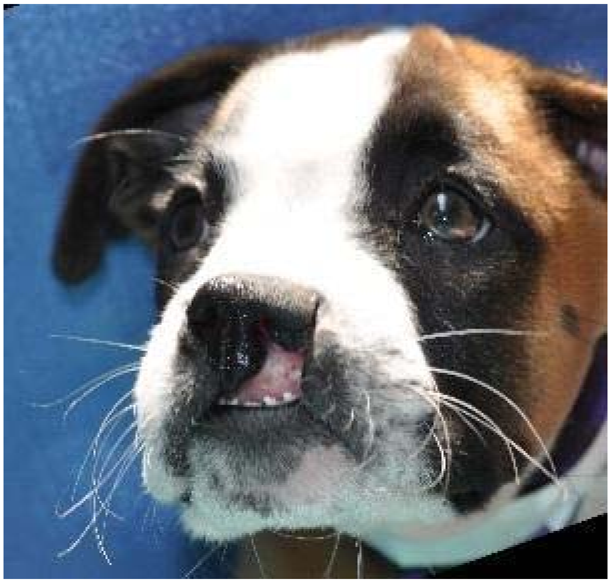

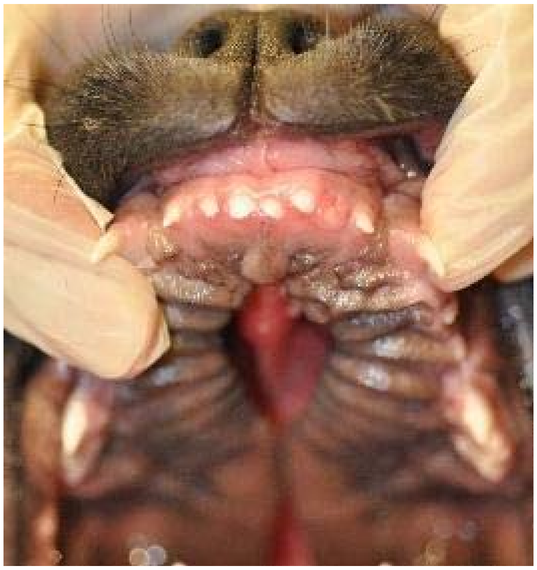

Section 7: Potential causes and costs

This study is concerned with the live-birth incidence of orofacial clefts. Please provide information pertaining to puppies born alive, rather than stillborn puppies.

Question 10: In your experience, were orofacial clefts associated with any particular perceived cause? If yes, what causes are suspected?

Question 11: What was the average size of litters with affected puppies (any type of orofacial cleft) in the past 12 months?

- 1
- 2 to 4
- 5 to 7
- 7 to 9
- 10 or more

Question 12: What was the average size of litters with no affected puppies in the past 12 months?

- 1
- 2 to 4
- 5 to 7
- 7 to 9
- 10 or more

Question 13: Please estimate in dollars the **typical** financial loss represented by **a single** live birth affected by a cleft defect.

Question 14: Please estimate in dollars the **total** financial loss represented by **all** live births affected by a cleft defect in the past 12 months.

Powered by Qualtrics
