# Supplementary material for: Incidence patterns of orofacial clefts in purebred dogs
Source: PLoS One. 2019 Nov 4;14(11):e0224574. doi: 10.1371/journal.pone.0224574 (PMC6827884; doi:10.1371/journal.pone.0224574)
Supplement: Attachment — Submitted filename: Point by point response.docx [file pone.0224574.s002.docx]

|  |  |  |  | |
| --- | --- | --- | --- | --- |
|  | Cornell University  College of Veterinary Medicine |  | | Santiago Peralta, DVM, Dipl. AVDC,  FF-AVDC-OMFS  *Assistant Professor, Dentistry and Oral Surgery*  C3-510 Veterinary Medical Center Ithaca, NY 14853-6401  telephone: 607.253.4387  fax: 607.253.3531  e-mail: sp888@cornell.edu  web: [www.vet.cornell.edu](http://www.vet.cornell.edu) |
|  |  |  |  |  |

**Response to reviewer comments:**

**Reviewer #1:** This article is the first broad epidemiological study of orofacial clefts in dogs, providing insights for future investigations regarding different aspects of this congenital abnormality.

The only changes required are as follows:

Lines 61 and 62 (caption): C) Intraoral view ...; D-E) Extraoral and intraoral views …

*Response*: Thank you very much for reviewing our work and noticing the error in the Figure 1 legend. We have revised the it accordingly.

**Reviewer #2:** This is a very interesting paper, which is well analysed and well written throughout. I have made a few very minor comments, but if the editor is happy, I am more than happy just to let him have a look at it when resubmitted and then he can accept it without me re-reviewing it. The comments are just minor grammatical things (due to the excellent nature of the manuscript). However, if you disagree please do not feel pressured to change them

Well done on producing a very good, interesting manuscript

*Response:* Many thanks for the thoughtful comments and meticulous review of our work.

Line 20 and 21- a comma maybe better than a semi colon

*Response:* Change made as suggested.

Line 29-30, a set of brackets may be better than hyphens

*Response:* Change made as suggested.

Line 41- you say useful models of diseases- maybe expand on which diseases?

*Response:* Change made as suggested.

Line 49- a comma, or brackets may be better than a hyphen

*Response:* Change made as suggested.

Line 86- a comma may work better than a semi-colon

*Response:* Change made as suggested.

Line 111- if a question queried information that could …etc- I am not quite sure that I follow this sentence so could it be reworded somehow?

*Response:* Change made as suggested.

Line 115- of instead of or maybe?

*Response:* Change made as suggested.

Line 121- no capital needed for internet

*Response:* We kept “Internet” based on the Merriam-Webster dictionary.

Line 156- maybe to determine if a biological interaction between ….

*Response:* Change made as suggested.

Line 167- if a significant statistical interaction …..

*Response:* Change made as suggested.

Line 191- maybe a new sentence instead of a semi colon?

*Response:* Change made as suggested.

Line 202- calculated may be better than tabulated for the incidence?

*Response:* Change made as suggested.

Line 205- I wondered if having the values in for the OR for the different breeds may help- but may make it less clear? I can leave that to your judgement

*Response:* Change made as suggested.

Lines 207-209- the line starting relatively few cases of …. Is a little unclear- maybe consider revising it?

*Response:* Change made as suggested.

Throughout- should breed names be capitalised?

*Response:* Change made as suggested.

Line 245- maybe a comma after interaction

*Response:* Change made as suggested.

Line 252- …incorporating sex found a statistically significant …..

*Response:* Change made as suggested.

Paragraph 277-285- you talk about rations in other studies, could you put an accurate one from your study in here?

*Response:* Change made as suggested.

Line 292- I am not a fan of using our, us, we etc in scientific writing

*Response:* Change made as suggested.

Line 306- does this fit with the evolutionary theory for the different dog breeds?

*Response:* Change made as suggested.

Line 341- which appear not to be (may sound better)

*Response:* Change made as suggested.

Line 342- on the other hand sounds a bit colloquial, maybe alternatively?

*Response:* Change made as suggested.

Paragraph 363- Is the geographic issue associated with breeds being associated with certain parts of the USA?

*Response:* Change made as suggested.
